# Stroke related to androgen deprivation therapy for prostate cancer: a meta-analysis and systematic review

**DOI:** 10.1186/s12885-016-2221-5

**Published:** 2016-03-03

**Authors:** Fanzheng Meng, Shimiao Zhu, Jinsheng Zhao, Larissa Vados, Lei Wang, Yusheng Zhao, Dan Zhao, Yuanjie Niu

**Affiliations:** Department of Neurology, Tianjin Nankai Hospital, Nankai Clinical School of Tianjin Medical University, Tianjin, China; Department of Urology, Second Hospital of Tianjin Medical Unversity, Tianjin Institute of Urology, 23 Pingjiang Road, Tianjin, 300211 China; Tianjin University of Traditional Chinese Medicine, Tianjin, China; Tianjin Institute of Medical and Pharmaceutical Sciences, Tianjin, China; First Teaching Hospital of Tianjin University of Traditional Chinese Medicine, Tianjin, China

**Keywords:** Stroke, Androgen deprivation therapy, Prostate cancer, Meta-analysis

## Abstract

**Background:**

Whether androgen deprivation therapy (ADT) leads to stroke morbidity is still unclear because of inconsistent evidence. We performed a systematic review and meta-analysis to evaluate if ADT used in men with prostate cancer (PCa) is associated with stroke.

**Methods and results:**

Medline, Embase and Cochrane Library databases up to September 30th 2014 were systematically searched with no date or language restriction, and reports from potentially relevant journals were complementally searched. Both randomized controlled trials and observational studies were included. Two reviewers independently extracted data and assessed study quality. Six observational studies finally met inclusion criteria, with 74,538 ADT users and 85,947 non-ADT users reporting stroke as an endpoint. Although no significant association was observed in pooled estimates, the incidence of stroke in ADT users was 12 % higher than control groups, (HR = 1.12, 95 % confidence interval [CI]: 0.95 to 1.32; *P* = 0.16). In subgroup-analyses of different ADT types, stroke was found to be significantly associated with gonadotropin-releasing hormone (GnRH) alone (HR = 1.20, 95 % CI: 1.12 to 1.28; *P* < 0.001), GnRH plus oral antiandrogen (AA) (HR = 1.23, 95 % CI: 1.13 to 1.34; *P* < 0.001) and orchiectomy (HR = 1.37, 95 % CI: 1.33 to 1. 46; *P* = 0.001), but not with AA alone (HR = 1.06, 95 % CI: 0.71 to 1.57; *P* = 0.78).

**Conclusions:**

GnRH alone, GnRH plus AA and orchiectomy is significantly associated with stroke in patients with PCa.

**Electronic supplementary material:**

The online version of this article (doi:10.1186/s12885-016-2221-5) contains supplementary material, which is available to authorized users.

## Background

Prostate cancer (PCa) is the most prevalent malignancy and remains a major healthcare problem in men in the United States [1]. Because the development and growth of PCa cells depends on androgens [2, 3], Androgen deprivation therapy (ADT) undoubtedly plays an important role to treat PCa, and recently, approximately 40 % of men diagnosed with PCa within 6 months have been treated with ADT in the US [4].

ADT is a palliative therapy, including different types of treatments such as gonadotropin-releasing hormone (GnRH), oral antiandrogen (AA), orchiectomy, and two or more types above combined. Although ADT is increasingly used as a treatment for PCa, this effect on prolonging life expectancy is unclear or even negative in several clinical studies [5, 6]. In our previous study [7], we found that ADT was positively associated with cardiovascular disease. Because both cardiovascular and cerebrovascular diseases share many common risk factors including atherosclerosis, dyslipidemia, visceral obesity, arterial endothelial dysfunction, and hypertension [8–12], ADT may also be associated with stroke. Additionally, one population-based cohort study [13] demonstrated that, GnRH agonists could significantly increase the risk of stroke (adjusted rate ratio [RR], 1.18; 95 % confidence interval [CI], 1.00–1.39). However, conflicting results were also reported. In a nation-wide population-based cohort study [14], authors found that ADT was associated with decreased stroke risk (adjusted hazard ratio [HR], 0.88; *P* = 0.001). Therefore, there is still no consensus regarding that ADT is associated with stroke.

Based on the controversy of this clinical issue, we performed a meta-analysis and systematic review to investigate whether ADT is associated with stroke in patients with PCa.

## Methods

### Search strategy and study selection

We systematically searched Medline, Embase and Cochrane Library databases up to September 30th 2014, with all possible combinations of the keywords as follows: *prostate cancer* or *prostate tumor* or *prostate carcinoma*, *androgen deprivation* or *androgen suppression* or *endocrine treatment* or *ADT* or *AST*; and *stroke* or *cerebrovascular* or *transient ischemic attack* or *hemiplegia* or *TIA* or *cardiovascular* (Additional file 1: Methods S1). No language, date, or other restrictions was used. Publications from potentially relevant journals were complementally searched.

Studies were included if they fulfilled the following inclusion criteria: 1) Patients diagnosed with PCa only; 2) Intervention groups must include ADT (either monotherapy or combination therapy); 3) Treatments in control groups were non-ADT (e.g. radical prostatectomy, radiotherapy, active surveillance.); 4) Studies must have the data of risk estimates with 95 % CIs; 5) Studies must report comparative data. If more than one study were identified from the same population, we extracted data from all available informations, rather than just a single publication.

### Data extraction and quality assessment

Two reviewers (Meng & Zhu) independently extracted the data from eligible and potentially relevant publications, with differences resolved by the third reviewer (Niu) as necessary. General characteristics of each included publication were recorded: first author’s name, year of publication, medical center, study design, sample size, population characteristics, follow-up period, interventions, definition of stroke morbidity, HRs and corresponding 95 % CIs of estimates in each comparisons. Definition of stroke was according to what descripted in each included publication. Our meta-analysis involved different types of ADT including AA, GnRH agonists, orchiectomy, and two or more types above combined.

Study qualities of the selected trials were assessed by the Jadad score [15]. Trails were considered to be of high quality if they achieved more than 4 scores. Newcastle-Ottawa quality assessment scale (NOS) [16] was used to assess the observational studies. Studies with more than 6 scores were considered high-quality. Two authors (Zhu & Meng) respectively addressed the assessments and discussed the discrepancies until agreement reached. Level of evidence (LOE) of all eligible publications were evaluated using the classifications of Phillips et al’s, [17].

### Subgroups analyses

In order to minimize the influence of concomitant treatments (e.g. radiotherapy and prostatectomy), subgroup analysis of ADT monotherapy vs watchful waiting or active surveillance (WW/AS) for stroke morbidity was carried out. ADT monotherapy was defined as a single therapeutic that in addition to ADT, no other previous therapy was used in intervention group. Considering the significance of existing heterogeneity in overall-analysis, additional subgroup-analyses for various types of ADT (e.g. GnRH, AA, GnRH + AA and Orchiectomy) vs non-ADT were also performed.

### Statistical analysis

Using the same methods as in our previous study [18], weighted HRs and 95 % CIs were estimate to compare all of these dichotomous variables. Different methods were employed to calculate the HRs on the basis of the data provided in the studies. When studies compared more than one types of ADT with the same control group severally (for example, GnRH vs Control, Orchiectomy vs Control), random effects meta-analyses were used to combine these results together as necessary.

Statistical heterogeneity among studies was evaluated with the Cochrane’s Q statistic [19]. In addition, inconsistency was quantified by *I*^2^ statistic (100 % × [(Q-df)/Q]), different *I*^2^ values (25, 50, and 75 %) denote different levels (low, medium, and high levels) of heterogeneity [20]. Using the Der-Simonian and Laird method, we chose random-effects models throughout this analysis no matter whether heterogeneity existed or not.

We used Begg adjusted rank correlation test and Egger linear regression test to evaluate publication bias. All meta-analyses were conducted with Review Manage (version 5.3; The Cochrane Collaboration, Oxford) and STATA software (version 11.0; College Station, Texas). Two-tailed *P* < 0.05 indicated significant difference statistically.

## Results

**Fig. 1:**
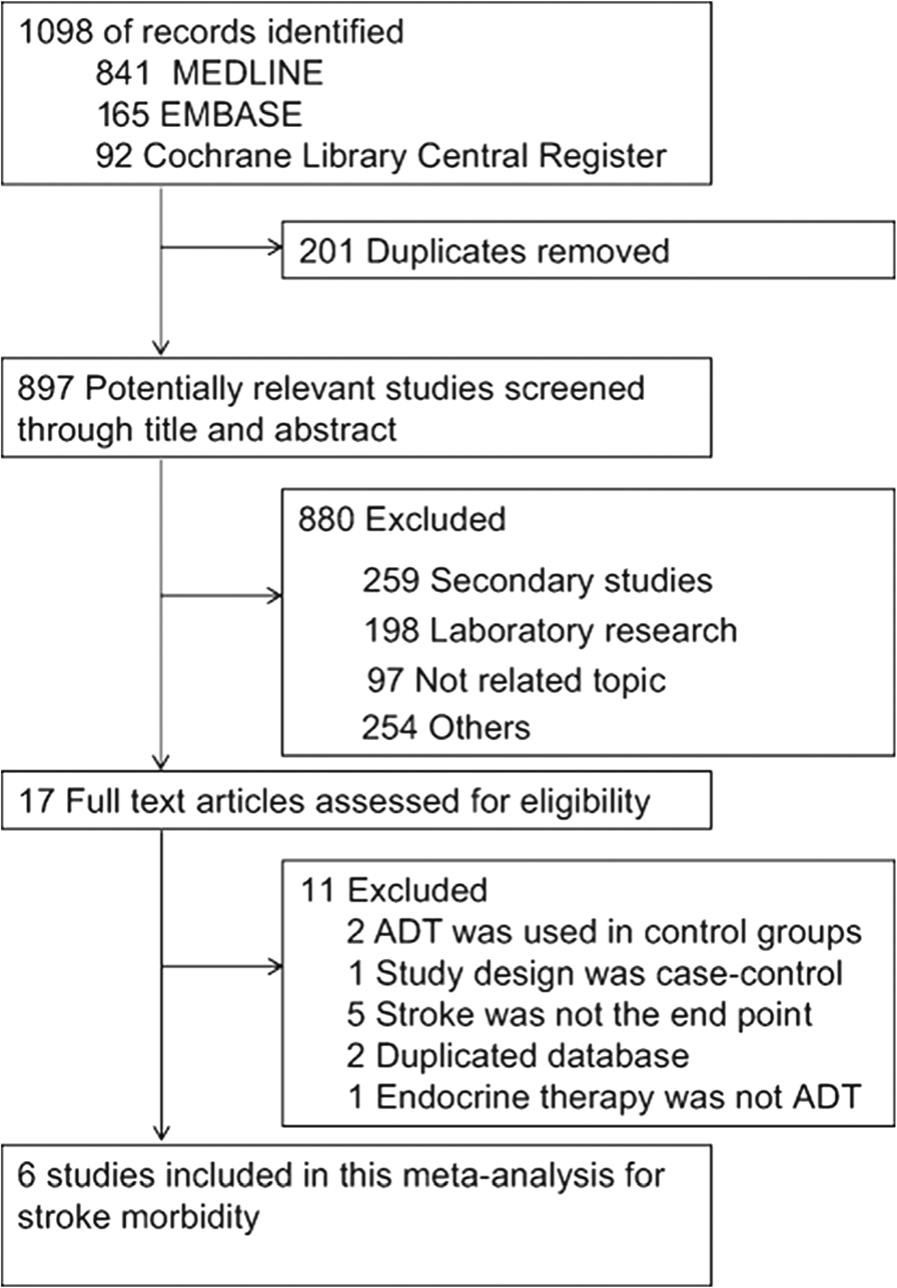
Flow Diagram of Search Strategy and Study Selection

Based on the titles, abstracts, and full text screening, we finally identified five cohort studies [14, 21–24] and one nested case–control study [13] that met the inclusion criteria. All articles included were published in English. Details of reasons for exclusion of articles through full text screening are shown in Additional file 1: Table S1. Figure 1 shows the literature search and study selection process of our meta-analysis.

### Study characteristics and study quality

HRs and 95 % CIs were directly given in two publications [14, 21], and four studies [13, 14, 23, 24] respectively compared different types of ADT with control groups. All of these observational studies were of high LOE (2a). Details of the eligible studies were summarized in Table 1. According to the assessment of NOS for observational studies, all eligible studies were high-quality with scores more than seven stars (Additional file 1: Table S2).

**Table 1:** Characteristics of Studies Investigating Stroke Related to ADT

First author year	Design, LOE	Database source (Duration)	Definition of Stroke (ICD codes)	Types of ADT	Treatments of control	No. of ADT/Control		Age y^a^(SD) of patients	Follow-up, (y^a^)		Hazard Ratios(95%CI)	
Jespersen et al. [25] 2013	Cohort, 2a	Danish Cancer Registry (2002–2010)	Ischemic Stroke/TIA (ICD-8 codes 433, 434.09/99, 436.01/436.90, ICD-10 codes DI63.x, DI64.x)	GnRH/AA	non-ADT	9204	20,307	71	3.3 (1.8 to 5.2)		1.19(1.06,1.35)^**c**^	1.17 (0.94, 1.50)^d^
				Orchiectomy		2060					1.11(0.90,1.36)^**c**^	
Hemelrijck et al. [24] 2010	Cohort, 2a	NPCR of Sweden (1997–2007)	Stroke (ICD-10: 160–164, G45)	GnRH agonist	RP	9066	26,432	≤65: 19,153	3.8	4.4	1.21(1.11,1.32)^b^	1.16 (1.01, 1.32)^d^
				AA		3391			4		0.88(0.76,1.00)^b^	
				GnRH + AA	WW/AS	11,646	19,527	66 to 74: 27,737	3.3	4.7	1.25(1.15,1.35)^b^	
				Orchiectomy		5340		≥75: 13,110	3.1		1.30(1.18,1.44)^b^	
				Other types		1199			-		-	
Alibhai et al. [14] 2009	Cohort, 2a	ICES (1995–2005)	Stroke (ICD-9-CM codes 430–438)	ADT	non-ADT	19,079/19,079		75 ± 6.3	6.47		0.88(0.81,0.96)^**c**^	
Keating et al. [23] 2010	Cohort, 2a	Veterans Healthcare Administration (2001–2004)	Ischemic Stroke/TIA (ICD-9 codes 433.XX −435.XX)	GnRH agonist	WW/AS	14,037	22,846	66.9 ± 8.6	2.6		1.18(1.02,1.36)^**c**^	1.18 (0.91, 1.51)^d^
				AA		1229					0.89(0.46,1.73)^**c**^	
				GnRH + AA		1838					0.91(0.60,1.39)^**c**^	
				Orchiectomy		308					1.81(1.15,2.84)^**c**^	
Huang et al. [21], 2014	Cohort, 2a	Queen Mary Hospital, Hong Kong (1998–2011)	Ischemic Stroke (NA)	ADT	non-ADT	517/228		72.2 ± 0.3	5.3		0.94 (0.35, 2.45)^c^	
Azoulay et al. [13] 2011	Nested Case–control, 2a	GPRD (1988–2008)	Stroke/TIA (NA)	GnRH agonist	non-ADT	3274	3960	72.3 ± 3.9	3.9		1.18(1.00,1.39)^**c**^	1.34 (1.15, 1.55)^d^
				AA		457					1.47(1.08,2.01)^**c**^	
				GnRH + AA		481					1.26(0.93,1.72)^**c**^	
				Orchiectomy		295					1.77(1.25,2.51)^**c**^	
				Other types		142					1.42(0.84,2.39)^**c**^	

Abbreviations: *LOE* level of evidence, *ADT* androgen deprivation therapy, *GnRH* gonadotropin-releasing hormone (leuteinizing hormone releasing hormone, LHRH), *AA* oral antiandrogens, *RP* radical prostatectomy/curative treatment, *WW/AS* watchful waiting (WW)/active surveillance (AS), *SD* standard deviation, *NA* not applicable, *NPCR* National Prostate Cancer Register, *GPRD* UK general practice research database, *ICES* institute for clinical evaluative sciences

^a^mean or median

^b^compared with WW/AS

^c^HR was directly given in the publication

^d^Combined estimates from all types of ADT with random effect meta-analysis

### Meta-analysis results

**Fig. 2:**
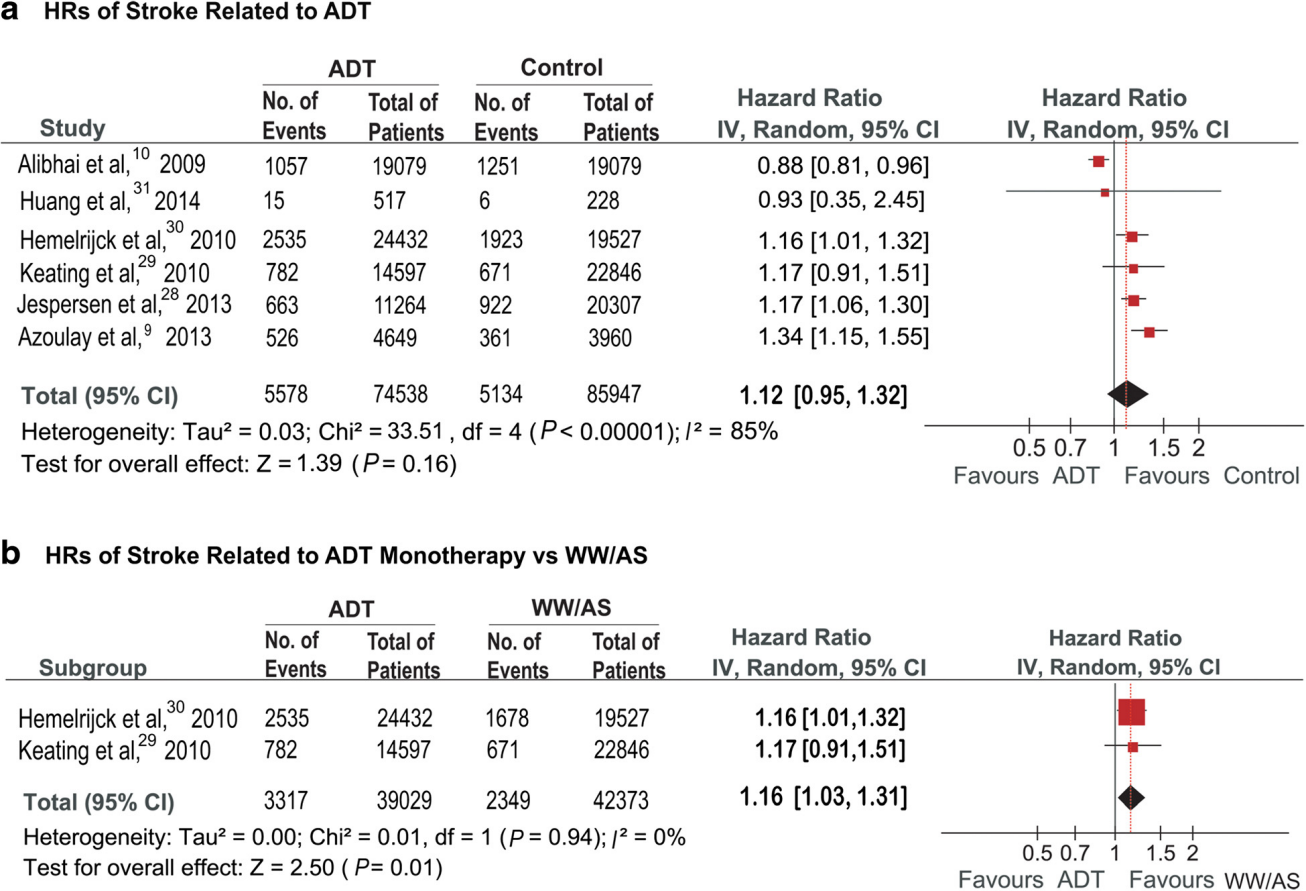
**a**. HRs of Stroke Related to ADT. **b**. HRs of Stroke Related to ADT Monotherapy vs WW/AS

**Fig. 3:**
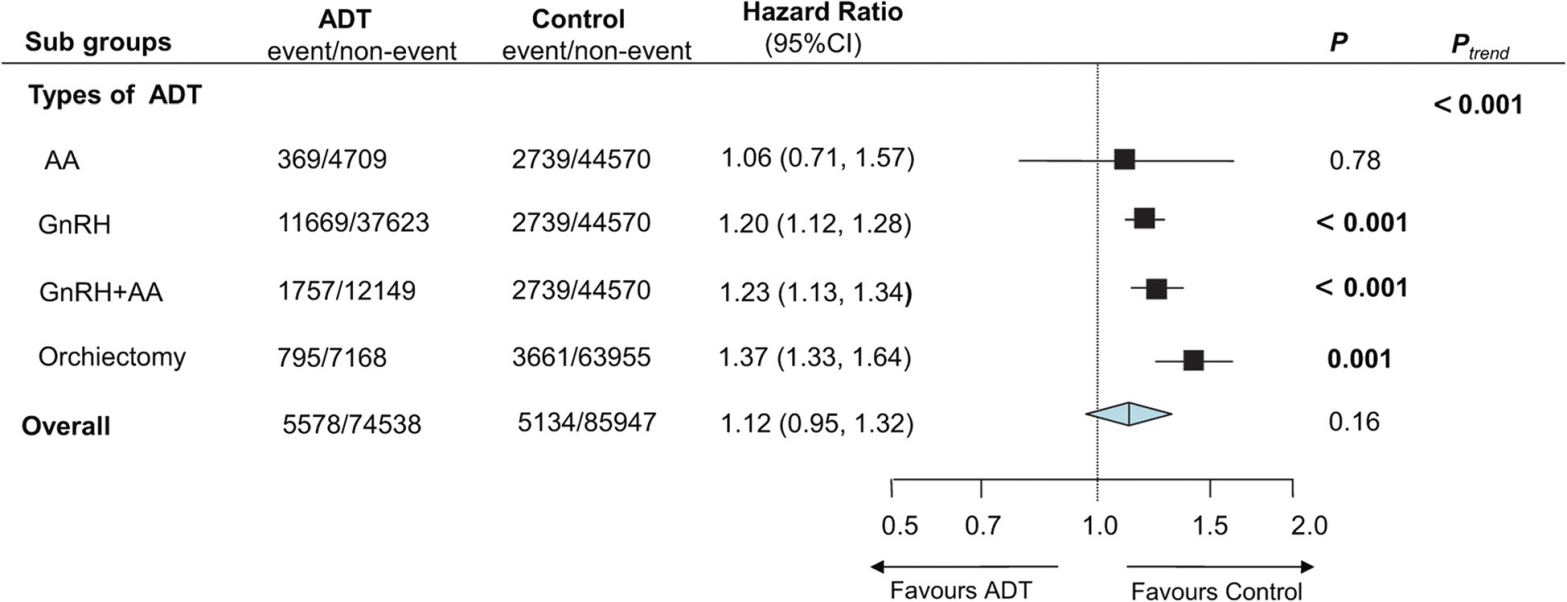
HRs of Subgroup Analyses for Stroke Related to Different Types of ADT

Six studies [13, 14, 21, 23–25] involving 160,485 participants were identified for inclusion criteria. Figure 2a showed the impact of ADT vs non-ADT on the end point of fatal or non-fatal stroke morbidity. 5578 (7.4 %) stroke events occurred among 74,538 ADT users compared with 5134 events (5.7 %) within control participants. Pooled HR showed that the incidence of stroke morbidity in ADT group was 12 % higher than non-ADT users, although statistically significant difference was not observed (HR = 1.12; 95 % CI, 0.95–1.32; *P* = 0.16). As to subgroup-analyses of different types of ADT, four studies [13, 23–25] were identified: three studies [13, 23, 24] respectively compared AA alone, GnRH alone and GnRH plus AA with control groups, four studies [13, 23–25] were available for the subgroup-analyses of orchiectomy vs non-ADT. Figure 3 showed the subgroup analyses for the effect of different types of ADT vs control on stroke events. Stroke was significantly associated with GnRH alone (HR = 1.20; 95 % CI 1.12–1.28; *P* < 0.001), GnRH plus AA (HR = 1.23; 95 % CI 1.13-1.34; *P* < 0.001), and orchiectomy (HR = 1.37; 95 % CI 1.33–1.64; *P* = 0.001), but not with AA alone (HR = 1.06; 95 % CI 0.71–1.57; *P* = 0.78). Details of meta-analyses for each type of ADT were shown in Additional file 1: Figure S1. Additionally, two studies [23, 24] with 81,402 patients were included for subgroup analysis of ADT monotherapy vs WW/AS. 6150 stroke events were recorded, containing 3317 events from ADT users (8.2 %) and 2349 from WW/AS groups (5.5 %). Pooled result revealed that ADT monotherapy could significantly increase the risk of stroke, with a higher incidence of 16 % than WW/AS (HR = 1.16, 95%CI: 1.03–1.31, *P* = 0.01; Fig. 2b).

## Discussion

Although the occurrence of stroke in men undergoing ADT with PCa has been an emerging problem over recent years, the relationship between ADT and stroke morbidity is still unclear. This meta-analysis including five population-based observational studies showed that ADT has a tendency to increase the risk of stroke. Evidence was directly proved by Azoulay et al. [13], showing that ADT could significantly increase the risk of stroke over a median follow-up of 3.9 years in men with newly diagnosed PCa (HR = 1.34, *P* = 0.0001). Another cohort study [24] involving 29,443 ADT users, and 19,527 with surveillance showed the standardized mortality ratios of stroke was 1.17.

ADT is considered to be effective when serum testosterone is declined to the recommended levels of 50 ng/dl, according to the 2012 NCCN (National Comprehensive Cancer Network) guidelines [26]. However, However, as reported in our previous study [7], low level of serum testosterone is likely related to many stroke risk factors including high triglyceride and low-density lipoprotein cholesterol levels, endothelial dysfunction and proinflammatory factors [12, 27–29]. In addition, previous studies [11, 30] showed that testosterone deficiency was significantly associated with hypertension, high body mass index, hypercoagulable states, and hyperfibrinogenemia [31]. All of these adverse effects may put patients at a high risk of stroke.

Out of the six studies we analyzed, only one [14] did not show the positive relationship between ADT and stroke (HR = 0.88; *P* = 0.001). This inconsistency was likely due to the contamination bias caused by radical prostatectomy. To reduce this bias, a sensitivity analysis was performed comparing ADT monotherapy with WW/AS. When ADT users undergoing other treatments were excluded, more significantly increased risk of stroke was observed in ADT monotherapy users (Fig. 2b).

There may be bias in the results due to different types of ADT that were used in some studies [13, 23–25]. Therefore, we carried out subgroup analyses stratified by different types of ADT in order to reduce this heterogeneity, and showed that stroke morbidity was significantly associated with GnRH alone, GnRH plus AA, and prostatectomy. The US Food and Drug Administration announced a safety warning that GnRH agonists could increase the risk of stroke in men receiving these drugs for treating PCa [1]. As previously reported [32], GnRH agonist may cause the development of metabolic syndrome, which in turn could accelerate the atherosclerotic process and then lead to increased stroke morbidity. One included cohort study [23] investigating the relationship between GnRH and stroke over a median follow-up of 2.6 years, concluded that GnRH was significantly associated with stroke morbidity (adjusted HR = 1.18, *P* = 0.03). All of these listed above was in accordance with our findings.

This meta-analysis and systematic review has several strengths. First, the included studies were all large-scale observational studies with long term of follow-up. Second, if the HRs were not available in eligible studies, all the data which could be used to calculate these were adjusted for the durations of follow-up. Finally, funnel plots showed balance in our assessment of publication bias. Begg’s and Egger’s tests also indicated that no significant publication bias existed (Table 2). Additionally, there was no obvious publication bias as shown in Additional file 1: Figure S2, since points are distributed around the verticals. Therefore, the findings in this meta-analysis can be considered credible.

**Table 2:** Pooled Results and Publication Bias for All Comparisons

Measurement	n^a^	Case/control	Heterogeneity		Pooled rate/HR	Begg’s test (*P*)	Egger’s test (*P*)
			*P*	*I* ^*2*^ (%)	(95 % CI)		
*Stroke morbidity*							
ADT vs Non-ADT	5	74538/85947	<0.001	85	1.13 (0.95–1.33)	0.806	0.261
AA vs Non-ADT	3	5078/47309	0.010	78	1.06 (0.71–1.57)	1.000	0.653
GnRH vs Non-ADT	3	49292/47309	0.930	0	1.20 (1.12–1.28)	1.000	0.125
GnRH plus AA vs Non-ADT	3	13906/47309	0.360	3	1.23 (1.13–1.34)	0.296	0.501
Orchiectomy vs Non-ADT	4	7963/67616	0.060	59	1.37 (1.33–1.64)	0.734	0.456

^a^ Number of included studies

However, we acknowledge that several limitations should be taken into consideration with the results found in this meta-analysis. First, all eligible reports were retrospective observational studies, which may introduce recall limitation, so the integrity of records may weaken the reliability of the results to some extent. Second, selection bias may have influenced our results. To minimize this bias, we carried out a predesigned search strategy with independent selection, and data was extracted by two reviewers. Third, incomplete data in some included publications [24, 25] may have influenced the overall result. As described in detail in our previous study [7], we have tried to minimize this limitation as much as possible. Furthermore, the stroke definition (ischemic, hemorrhagic, or TIA) was not specified in some studies [13, 14, 24], introducing potential bias in stroke incidence estimate. However, most of events in these eligible studies were defined as ischemic events, and this bias is possibly minimized because these overall stroke rates were similar to the study [23] only including ischemic events as the endpoint. Finally, the certain characteristics of patients that may contribute to stroke were different in each included study, which might confound the presented results. Therefore, adjusted data were extracted when available to minimize the bias.

## Conclusion

In conclusion, there is a tendency that ADT could increase the risk of stroke. Significant association of ADT monotherapy with stroke was observed after removing patients with prostatectomy and radiotherapy. Additionally, GnRH, GnRH plus AA, and orchiectomy can significantly result in stroke. These findings may help clinicians be aware of the potential risks of ADT and ensure clinical management when prescribing this treatment. Additional studies should also focus on the different definitions of stroke since they require different approaches to treatment.

## Additional file

Additional file 1:
**Methods S1.** Literature Search Strategy. **Table S1.** List of Excluded Full-text Articles with Reasons for Exclusions. **Table S2.** Newcastle-Ottawa Scale Quality Assessment of Included Studies. **Figure S1.** Details of Subgroup Analyses for Stroke Related to Different Types of ADT. **Figure S2.** Funnel plots for Meta-analyses. (DOC 180 kb)
