# Supplementary material for: Stroke related to androgen deprivation therapy for prostate cancer: a meta-analysis and systematic review
Source: BMC Cancer. 2016 Mar 3;16:180. doi: 10.1186/s12885-016-2221-5 (PMC4778362; doi:10.1186/s12885-016-2221-5)

**Methods S1:** **Literature Search Strategy**

Search date: September 30^th^ 2014

Databases searched: MEDLINE, EMBASE, and Cochrane Library Central Register.

**1. Database: MEDLINE**

Search Strategy:

| #1 | Search prostate cancer[MeSH] OR prostate tumor[MeSH] OR prostate carcinoma[MeSH] Field: Full text | 124,091 |
| --- | --- | --- |
| #2 | Search androgen deprivation[MeSH] OR androgen suppression [MeSH] OR endocrine treatment OR ADT[MeSH] OR AST[MeSH] Field: Full text | 176,031 |
| #3 | Search #1 AND #2 Field: Full text | 7,630 |
| #4 | Search cardiovascular [MeSH] OR stroke [MeSH] OR cerebrovascular [MeSH] OR transient ischemic attack [MeSH] OR hemiplegia [MeSH] OR TIA[MeSH] Field: Full text | 1603,639 |
| #5 | Search #3 AND #4 Field: Full text | 841 |

**2. Database: EMBASE**

Search Strategy:

| #1 | **'prostate'**/exp AND **'cancer'**/exp OR **'prostate'**/exp AND **'tumor'**/exp OR **'prostate'**/exp AND **'carcinoma'**/exp AND [humans]/lim AND[abstracts]/lim | 3,253 |
| --- | --- | --- |
| #2 | **'androgen'**/exp AND **deprivation** OR (**'androgen'**/exp AND **suppression**) OR (**endocrine** AND **treatment**)OR**ADT** OR **AST** AND[humans]/limAND[full text]/lim | 801,724 |
| #3 | #1 AND #2 Not animals full text. | 416 |
| #4 | **'stroke'**/expOR **cerebrovascular** OR **cardiovascular** OR **transient** AND **ischemic** AND  **attack** OR **'hemiplegia'**/exp OR **'TIA'**/exp AND [humans]/lim AND [full text]/lim | 32,500 |
| #5 | #3 AND #4 full text. Not animals | 165 |

**3. Database: The Cochrane Library Central Register**

Search Strategy:

| #1 | prostate cancer or prostate tumor or prostate carcinoma: all text, kw (Word variations have been searched) | 6,511 |
| --- | --- | --- |
| #2 | androgen deprivation or androgen suppression or endocrine treatment or ADT or AST: all text kw (Word variations have been searched) | 5,291 |
| #3 | #1 AND #2 all text | 681 |
| #4 | stroke or cardiovascular or cerebrovascular or transient ischemic attack or hemiplegia or TIA all text kw (Word variations have been searched) | 72,454 |
| #5 | #3 AND #4 all text | 92 |

**Table S1.** List of Excluded Full-text Articles with Reasons for Exclusions

|  |  | Reason for exclusion |
| --- | --- | --- |
| 1 | Bruchovsky N, Klotz L, Crook J, Phillips N, Abersbach J, Goldenberg SL. Quality of life, morbidity, and mortality results of a prospective phase II study of intermittent androgen suppression for men with evidence of prostate-specific antigen relapse after radiation therapy for locally advanced prostate cancer. Clinical genitourinary cancer. 2008;6(1):46-52. | All the patients received ADT |
| 2 | Hayes JH, Chen MH, Moran BJ, Braccioforte MH, Dosoretz DE, Salenius S, et al. Androgen-suppression therapy for prostate cancer and the risk of death in men with a history of myocardial infarction or stroke. BJU international. 2010; 106(7):979-85. | Stroke was not the endpoint |
| 3 | Grant JD, Litwin MS, Kwan L, Lee SP, Steinberg ML, King CR. Does hormone therapy exacerbate the adverse effects of radiotherapy in men with prostate cancer? A quality of life study. The Journal of urology. 2011;185(5):1674-80. | Stroke was not the endpoint |
| 4 | Smith MR, Klotz L, van der Meulen E, Colli E, Tanko LB. Gonadotropin-releasing hormone blockers and cardiovascular disease risk: analysis of prospective clinical trials of degarelix. The Journal of urology. 2011;186(5):1835-42. | Stroke was not the endpoint |
| 5 | Nguyen PL, Chen MH, Hoffman KE, Chen RC, Hu JC, Bennett CL, et al. Cardiovascular comorbidity and treatment regret in men with recurrent prostate cancer. BJU international. 2012; 110(2):201-5. | Stroke was not the endpoint |
| 6 | Kumamoto Y, Tsukamoto T, Umehara T, et al. [Clinical studies on endocrine therapy of prostatic carcinoma (2): Prognosis of patients with prostatic carcinoma given endocrine therapy, and analyses of causes of death and side effects of endocrine therapy]. *Hinyokika kiyo. Acta urologica Japonica.* Mar 1990;36(3):285-293. | Endocrine therapy was not ADT |
| 7 | Keating NL, O'Malley AJ, Freedland SJ, Smith MR. Does comorbidity influence the risk of myocardial infarction or diabetes during androgen-deprivation therapy for prostate cancer? European urology. 2013;64(1):159-66. | Stroke was not the endpoint |
| 8 | Van Hemelrijck M, Garmo H, Holmberg L, Stattin P, Adolfsson J. Multiple events of fractures and cardiovascular and thromboembolic disease following prostate cancer diagnosis: results from the population-based PCBaSe Sweden. European urology. 2012; 61(4):690-700. | Duplicate – completed with  Hemelrijck et al. 2010 |
| 9 | Robinson D, Garmo H, Lindahl B, Van Hemelrijck M, Adolfsson J, Bratt O, et al. Ischemic heart disease and stroke before and during endocrine treatment for prostate cancer in PCBaSe Sweden. International journal of cancer Journal international du cancer. 2012;130(2):478-87. | Duplicate – completed with  Hemelrijck et al. 2010 |
| 10 | Chung SD, Chen YK, Wu FJ, Lin HC. Hormone therapy for prostate cancer and the risk of stroke: a 5-year follow-up study. *BJU international.* Apr 2012;109(7):1001-1005. | Study design was case-control study |
| 11 | Langley RE, Cafferty FH, Alhasso AA, Rosen SD, Sundaram SK, Freeman SC, Pollock P, Jinks RC, Godsland IF, Kockelbergh R et al: Cardiovascular outcomes in patients with locally advanced and metastatic prostate cancer treated with luteinising-hormone-releasing-hormone agonists or transdermal oestrogen: the randomised, phase 2 MRC PATCH trial (PR09). The lancet oncology 2013, 14(4):306-316. | All the patients received ADT |

**Table S2.** Newcastle-Ottawa Scale Quality Assessment of Included Studies

| **study** | **Selection** | | | | **Comparability** | **Outcome** | | | **Scores** |
| --- | --- | --- | --- | --- | --- | --- | --- | --- | --- |
|  | Representativ-eness of exposed cohort | Selection of non-expos-ed cohort | Ascertainment of exposure | outcome of interest was not present at  start of study | Comparability on the basis of the design or analysis^a^ | Assessment  of outcome | follow-up long enough for outcomes to occur | Adequacy of  follow up  of cohorts |  |
| Jespersen et al, ^28^ 2013 | ☆ | ☆ | ☆ | ☆ | ☆ | ☆ | ☆ | ☆ | 8 |
| Hemelrijck et al, ^30^ 2010 | ☆ | ☆ | ☆ | ☆ | ☆ | ☆ | ☆ | ☆ | 8 |
| Alibhai et al, ^10^ 2009 | ☆ | ☆ | ☆ | ☆ | ☆☆ | ☆ | ☆ | ☆ | 9 |
| Keating et al, ^29^ 2010 | - | ☆ | ☆ | ☆ | ☆ | ☆ | ☆ | ☆ | 7 |
| Azoulay et al, ^9^ 2011 | ☆ | ☆ | ☆ | ☆ | ☆☆ | - | ☆ | ☆ | 8 |
| Huang et al,^31^ 2014 | ☆ | ☆ | ☆ | ☆ | ☆ | - | ☆ | ☆ | 7 |

^a^ A maximum of 2 stars can be allotted in this category, one for the most important factors (Age) the other for second important factors (gender, race, etc.).

**Figure S1.** Details of Subgroup Analyses for Stroke Related to Different Types of ADT


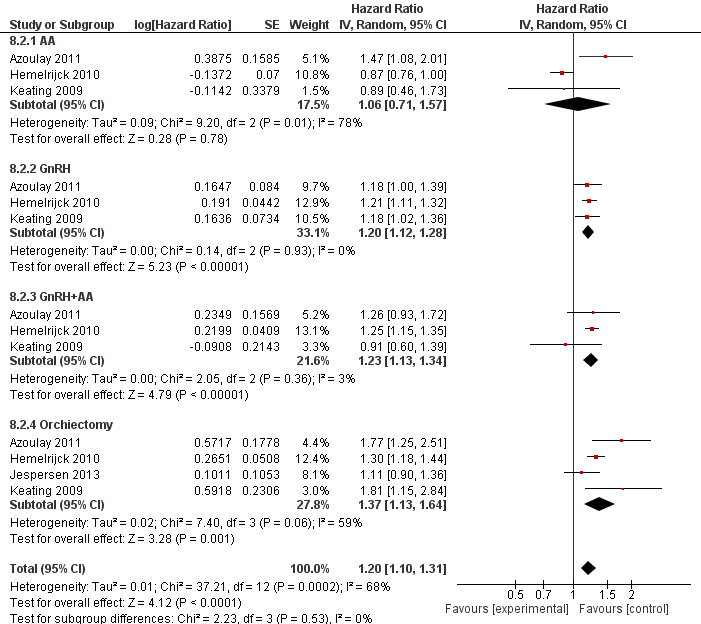


**Figure S2**. Funnel plots for Meta-analyses


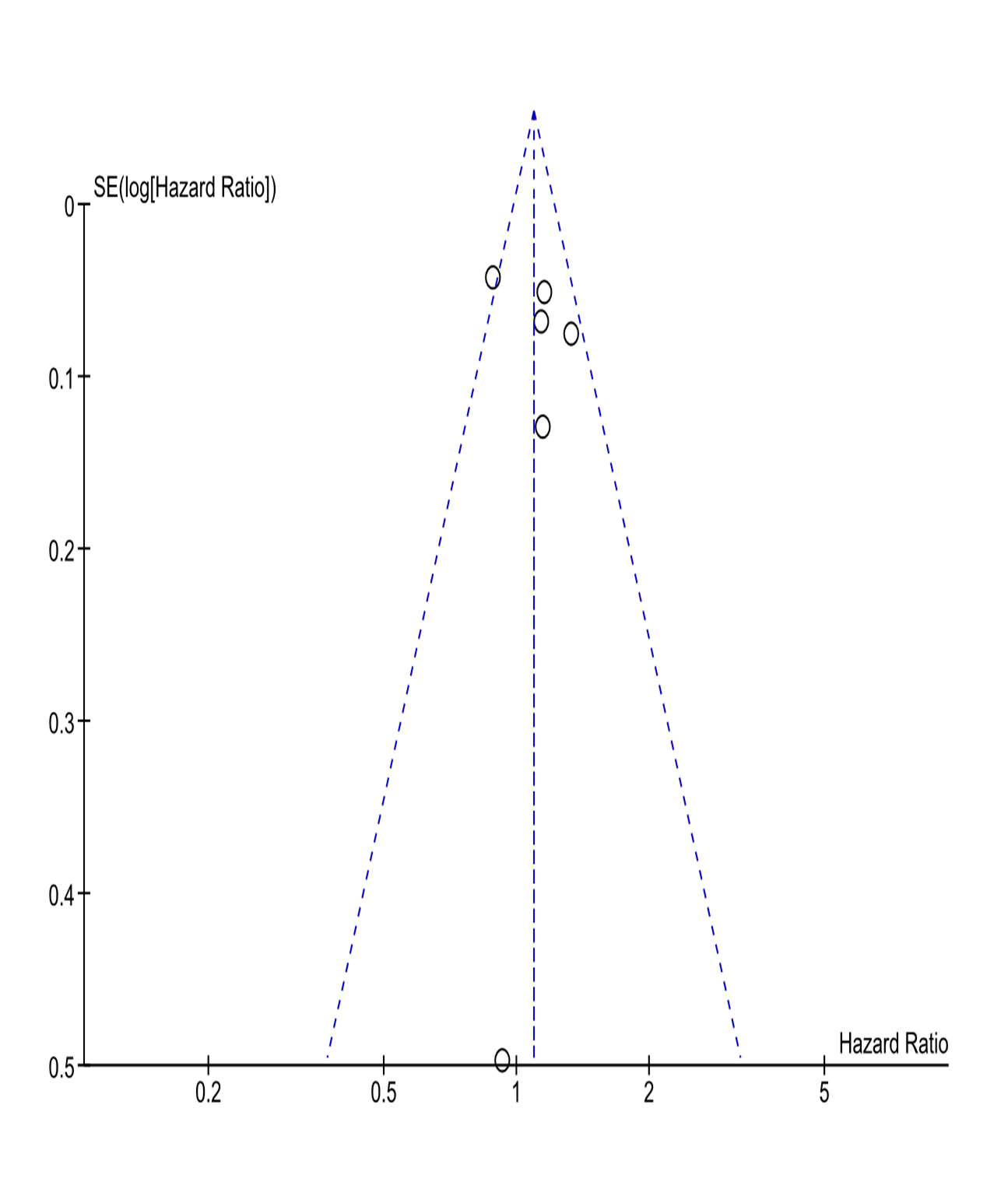

Supplement: Additional file 1: — Methods S1. Literature Search Strategy. Table S1. List of Excluded Full-text Articles with Reasons for Exclusions. Table S2. Newcastle-Ottawa Scale Quality Assessment of Included Studies. Figure S1. Details of Subgroup Analyses for Stroke Related to Different Types of ADT. Figure S2. Funnel plots for Meta-analyses. (DOC 180 kb) [file 12885_2016_2221_MOESM1_ESM.doc]
